# Notes on the Treatment of Eclampsia

**Published:** 1930

**Authors:** G. H. Temple

**Affiliations:** Senior Surgeon, Weston-super-Mare General Hospital


### NOTES ON THE TREATMENT OF ECLAMPSIA.

BY

G. H. TEMPLE, M.B., C.M.,

*Senior Surgeon, Weston-super-Mare General Hospital.*

---

THE cause of eclampsia appears to be as far from solution as it was forty years ago, and all that can be stated is that we have to deal with a toxæmia, the result of pregnancy, so all treatment aims at the elimination of the poison and termination of the cause at the best time. We may suppose, as a working hypothesis, that some substance is normally secreted during pregnancy, perhaps giving rise to the early vomiting, which, in excess, becomes a poison. Eclampsia is less common to-day than formerly, which may fairly be attributed to the growth of ante-natal care and suitable treatment of the known danger signals, yet in an analysis of all British cases Eden finds :—

1. The general mortality is unnecessarily high
2. The best results are obtained in Dublin.
3. The mortality is low in mild and high in severe cases, irrespective of the method of treatment.
4. In both groups the highest death-rate occurs in cæsarean section and *accouchement forcé*.

The main lines of treatment have been styled conservative and radical. The first was the usual procedure at the end of the last century, and gave a mortality of 20 to 28 per cent. ; then came the radical period, which reduced the mortality to about 18 per cent. ; but during the last five years a combination of the two has further improved the statistics.

Stroganoff's method consists of the administration of morphia gr. $\frac{1}{4}$, an hour later 30 grs. chloral in 200 c.c. saline per rectum or in milk by the mouth ; in three hours the morphia is repeated, in seven hours 30 grs. chloral, with two more doses of 20 grs. at thirteen and twenty-one hours. He uses chloroform to control the convulsions, but if more than three occur 400 c.c. blood are withdrawn. Delivery is not effected till the os is dilated. His mortality covering 2,208 cases is 9·8 per cent.

The Dublin technique consists of starvation, stomach and bowel lavage—mag. sulph. in the stomach, sod. bicarb. in the intestine—subcutaneous infusion of sod. bicarb. solution, and delivery when the os is dilated. Neither morphia, chloroform, chloral nor venesection is used.

Williams, of Johns Hopkins Hospital, advises treating the patient in a quiet, dark room, giving morphia gr. $\frac{1}{4}$, which is repeated in an hour, but not more than gr. $\frac{1}{2}$ in twenty-four hours. If a second convulsion occurs he bleeds to 1,000 c.c. or until the blood pressure falls to 100, irrespective of the pulse-rate. A large amount of water should be given to drink if possible, or 500 c.c. of 5 per cent. glucose injected into a vein, which is repeated in twelve hours. The uterus is not emptied till the os is fully dilated, but if the convulsions or coma continue and the cervix is dilated to 5 cm. then manual dilatation is used and delivery effected. Cæsarean section is distinctly contra-indicated except when required for other conditions. Williams prefers ether or gas and oxygen for the anæsthetic.

Bleeding must be full to be efficacious, and occasionally results in complete disappearance of symptoms. Lichtenstein also upholds free bleeding.

Queen Charlotte's Hospital follows the Dublin treatment, but bleeding is practised for cyanosis, full pulse and cardiac dilatation. Obstetric interference is not favoured, and yet the lowest mortality occurs in induced labour. The Edinburgh Maternity Hospital follows much the same technique, with cæsarean section in fulminating cases. The mortality is relatively high, and has been analysed in a number of very useful tables in the *Transactions of the Edinburgh Obstetrical Society*, 20th April, 1929. The highest mortality at this hospital occurs in inverse ratio to the blood-pressure, but is very high where the pulse and temperature were respectively over 120 and 102°.

The writer's routine has been to empty the uterus as soon as possible. The stomach is washed out with weak solution of potassium permanganate till it returns pink. This is followed by the introduction of 1 pint of tea with milk and 1 oz. of castor oil through the tube. Venesection is a routine if the case is at all severe, 20 to 30 oz. in amount, with saline infusion in every case. In fulminating cases where manual dilatation and forceps was not possible *accouchement forcé* carefully performed has never been regretted. The anæsthetic has been chloroform, and no other drug has been used. In this series of 48 cases the mortality was 4·17 per cent., there were 34 consecutive recoveries, of which 9 were delivered by Bossi's method without mishap.

### CONCLUSIONS.

Emerging from the mass of statistics, it seems that conservative methods are best in mild cases, but that similar treatment in severe cases is uniformly bad.

Termination of labour is the main objective; bleeding is thought well of on all hands, while stomach lavage and kidney stimulation are considered necessary, but drugs are of doubtful value. Chloroform is safe and probably better than ether.

Cæsarean section is only necessary when other reasons than eclampsia are present.

Sweating is of no value, perhaps harmful, and saline should be replaced by glucose, as there is already an excess of chlorides. Intravenous magnesium sulphate is strongly advocated by Davidson as a sedative with marked effect on the elimination by the kidneys.

The urgent danger signs are coma, rapid pulse, high temperature and urine solid with albumen.

The premonitory signs of eclampsia are well known, such as œdema which persists after rest, headache with albuminuria, blurring of vision, persistent vomiting (which has been noted at Queen Charlotte's in 50 per cent. of the cases), abnormal increase of body weight. Albuminuria is invariably present at some time before an attack.

If the patient does not improve under treatment induction of labour is worth considering, but if any of the symptoms show a tendency to increase, or fresh signs arise, such as severe headache, jaundice, urgent vomiting, marked increase of albumen or suppression of urine, induction is imperative. Some varieties of toxæmia are so dangerous that no time should be wasted on expectant treatment.

